# Bromocriptine enhances the uptake of ^99m^Tc-MIBI in patients with hepatocellular carcinoma

**DOI:** 10.7555/JBR.26.20110075

**Published:** 2012-04-12

**Authors:** Xiangting Chai, Qiaoyu Liu, Wenyu Shao, Feng Zhang, Xuehao Wang, Hai Wang

**Affiliations:** a Department of Internal Medicine, Jiaonan People's Hospital of Shandong Province, Jiaonan, Shandong 266400, China;; b Department of General Surgery, the First Affiliated Hospital of Nanjing Medical University, Nanjing, Jiangsu 210029, China.

**Keywords:** liver cancer, multidrug resistance, *P*-glycoprotein, ^99m^Tc-MIBI, bromocriptine

## Abstract

^99m^Tc-methoxyisobutyl isonitrile (MIBI) is a suitable transport substrate for the multidrug resistance gene product *P*-glycoprotein (*P*-gp) and widely used for tumor imaging. Bromocriptine has been shown to inhibit the ATPase activity and the function of *P*-gp. We hypothesized that bromocriptine could promote the accumulation of MIBI by inhibiting *P*-gp activities, a feature that can be taken advantage of for enhancing ^99m^Tc-MIBI imaging. In the current study, we sought to investigate whether bromocriptine enhanced the uptake of ^99m^Tc-MIBI in hepatocellular carcinoma patients. Sixty primary hepatocellular carcinoma patients received ^99m^Tc-MIBI single photon emission computer tomgraphy (SPECT) prior to surgery. ^99m^Tc-MIBI SPECT was performed 15 and 120 min after injection of 20 mCi ^99m^Tc-MIBI, and early uptake, delayed uptake (L/Nd), and washout rate (L/Nwr) of ^99m^Tc-MIBI were obtained. In addition, a second ^99m^Tc-MIBI SPECT was performed according to the same method 48 h after bromocriptine administration. We found that, prior to bromocriptine administration, significant MIBI uptake in tumor lesions was noted in only 10 (16.7%, 10/60) patients with hepatocellular carcinoma. No significant MIBI uptake was observed in the tumor lesions of the remaining 50 (83.3%, 50/60) hepatocellular carcinoma patients. Following bromocriptine administration, all the patients without apparent MIBI uptake demonstrated significant MIBI uptake on ^99m^Tc-MIBI SPECT (*P* < 0.05). Our findings indicate that bromocriptine enhances the uptake of ^99m^Tc-MIBI in patients with hepatocellular carcinoma.

## INTRODUCTION

Until now, the main obstacle to a successful cure of cancer has been the intrinsic or acquired resistance of the neoplastic cells to a variety of structurally and functionally heterogeneous anticancer agents, which is named multidrug resistance (MDR). *P*-glycoprotein (*P*-gp) is a multidrug resistance gene product that is found on the surface of multidrug resistant cancer cells and is implicated in multidrug resistance[1]-[2]. As an energy dependent drug efflux pump, the protein reduces the accumulation of chemotherapeutic drugs in multidrug resistant cells.[3] Therefore, a key step in overcoming multidrug resistance is by inhibition of multidrug resistance-1 (MDR1)/*P*-gp expression in hepatocellular carcinoma cells. Various approaches have been attempted to block MDR1 overexpression[4]-[6].

Multidrug resistance modulators capable of blocking *P*-gp-mediated drug efflux have been suggested to reverse *P*-gp-mediated drug resistance and to improve the outcome of cancer chemotherapy. They include the anti-arrhythmic drug verapamil and the immunosuppressant cyclosporine, and the second-generation multidrug resistance modulators PSC833 and MS209. However, clinical application of these drugs is hampered by their side effects and the possibility of inhibiting other transporters that are not related to multidrug resistance.

^99m^Tc-methoxyisobutyl isonitrile (MIBI) is a cationic lipophilic agent and widely used for myocardial perfusion imaging and detection of various tumors[10]-[16]. Recent studies have revealed that ^99m^Tc-MIBI is a suitable transport substrate for *P*-gp and thus may provide additional information about the *P*-gp status of tumor cells[17],[18]. MIBI is accumulated within the mitochondria and cytoplasm and malignant tumors show increased transmembrane potential as a result of increased metabolic requirements that induce increased accumulation of MIBI[19]. ^99m^Tc-MIBI imaging has the advantage of noninvasively detecting the presence of *P*-gp overexpression *in vivo*[20]-[21]. In our previous study, we have found that ^99m^Tc-MIBI is useful for noninvasively detecting the expression of *P*-gp in hepatocellular carcinoma[22].

Bromocriptine is a hydrophobic polycyclic molecule and a D_2_ dopaminergic receptor agonist and has previously been reported to inhibit the ATPase activity and the function of *P*-gp[7]. The drug has been used to treat hyperprolactinemia, acromegaly and Parkinson's disease for more than two decades and is associated with slight side effects[8],[9]. However, there has been no report on the use of bromocriptine in reversing multidrug resistance in cancer in humans. We speculate that bromocriptine could promote the accumulation of MIBI, a transport substrate for *P*-gp, by inhibiting *P*-gp activities, a feature that can be taken advantage of for enhancing ^99m^Tc-MIBI imaging. In the current study, we compared the visualization rate by ^99m^Tc-MIBI imaging of hepatocellular carcinoma patients before and following administration of bromocriptine.

## SUBJECTS AND METHODS

### Subjects

We reviewed the clinicopathological and radiological data of consecutive chemotherapy-naïve patients with pathologically proven hepatocellular carcinoma who sought surgical treatment between January 2006 and December 2009 at the Hepatic Center of Tongji Hospital, Tongji Medical College, Huazhong University of Science and Technology, Wuhan, China. All patients underwent physical examination, abdominal ultrasonography and computerized tomography (CT). The protocol was approved by the local institutional review board and patient consent was not required because of the retrospective nature of the study. All patients underwent ^99m^Tc-MIBI SPECT prior to surgery.

### ^99m^Tc-MIBI SPECT

Liver imaging was performed with a double-head gamma camera equipped with a high-resolution parallel-hole collimator (PRISM 2000; Marconi Medical Systems, Cleveland, OH, USA). Images were obtained 15 and 120 min after injection of 20 mCi ^99m^Tc-MIBI. Early and delayed SPECT of the liver was performed in all patients. After the first liver SPECT, patients were required to take 2.5 mg bromocriptine (Novartis, Basel, Switzerland) orally 3 times per d for 3 d. Thereafter, the second liver SPECT was performed. For SPECT of the liver, 72 projections were obtained using a 64×64 matrix at 45 s per view. Image reconstruction was performed using filtered back projection with Butter-worth and ramp filters. Transverse, coronal, and sagittal sections were reconstructed. Attenuation correction was not applied. SPECT images were compared with liver CT images, and accumulation in liver tumors was interpreted independently by two nuclear medicine physicians who were blind to the clinicopathological data of patients. Disagreements were resolved by consensus, with a third observer as referee. The findings on ^99m^Tc-MIBI liver imaging were evaluated semi-quantitatively. Regions of interest (ROIs) were manually defined on the transaxial tomograms with the lesion's highest uptake in the middle of the tumor. The ROIs placed on the lesions (L) encompassed all pixels that had uptake values of >90% of the maximum uptake in that slice, and the average counting rate in each ROI was calculated. Another ROI of the same size was then drawn over the normal liver (N) on the same transverse section. The early uptake (L/Ne) and the delayed uptake (L/Nd) were obtained. The washout rate (L/Nwr) was calculated using the following formula: L/Nwr = (L/Ne-L/Nd)×100 (L/Ne).

### Statistical analysis

The data for L/Ne, L/Nd, and L/Nwr was expressed as mean±SD. Student's *t* test was used to evaluate the significance of differences. A *P* value of 0.05 or less was considered to be significant.

## RESULTS

### Patient demographic and disease characteristics

Sixty patients with hepatocellular carcinoma were included in the study, including 24 female and 36 male patients with a mean age of 60±11.5 years (range, 30-73 years). Fifty-two (86.7%, 52/60) patients were hepatitis B surface antigen positive, 3 (5.0%, 3/60) were anti-hepatitis C virus antibody positive, and the remaining patients had no known cause of hepatocellular carcinoma. The tumor size of these patients ranged from 1.5 to 15.0 cm.

### Bromocriptine enhances the uptake of ^99m^Tc-MIBI in patients with hepatocellular carcinoma

**Fig. 1:**
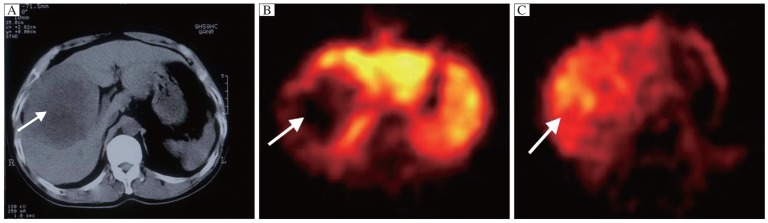
MRI and SPECT detection of hepatocellular carcinoma in the right liver. A: MRI image showed that the tumor was located in the posterior lobe of the right liver. B: ^99m^Tc-MIBI SPECT revealed that MIBI accumulated densely at tumor lesion. C: Significant MIBI uptake on ^99m^Tc-MIBI SPECT was noted in tumor lesion after intake of bromocriptine. Arrow indicates the site of tumor lesion.

Analysis of ^99m^Tc-MIBI SPECT imaging data of these hepatocellular carcinoma patients revealed that, prior to bromocriptine administration, significant MIBI uptake in tumor lesions was noted in only 10 (16.7%, 10/60) patients with hepatocellular carcinoma. No significant MIBI uptake was observed in the tumor lesions of the remaining 50 (83.3% 50/60) hepatocellular carcinoma patients. Following bromocriptine administration, all the patients without apparent MIBI uptake demonstrated significant MIBI uptake on ^99m^Tc-MIBI SPECT (*P* < 0.05) (***Fig. 1***).

## DISCUSSION

*P*-gp, as a drug efflux pump, extrudes ^99m^Tc-MIBI and other drugs from the cells[23],[24]. In animal models and clinical studies, faster clearance of ^99m^Tc-MIBI was observed in tumors that expressed *P*-gp than those that did not[25]-[27]. It was also found that ^99m^Tc-MIBI L/Nwr values from treatment naïve breast cancers overexpressing *P*-gp were 2.7 times higher than those not expressing *P*-gp[28]. In our earlier study, we have found that *P*-gp expression was significantly higher in those patients with no apparent ^99m^Tc-MIBI uptake compared with those with significant uptake[22].

There have been numerous attempts to restore chemosensitivity to various chemotherapeutic drugs in recalcitrant cancer cells with MDR[29]-[31]. These strategies have so far remained unsuccessful because of considerable side effect. Bromocriptine, a classical D_2_ dopaminergic receptor agonist, has been reported to inhibit the ATPase activity and the function of *P*-gp[7]. The drug has been used to treat hyperprolactinemia and Parkinson's disease and has exhibited mild side effects[32]-[35]. Our previous results showed that bromocriptine blocked *P*-gp-mediated drug resistance in HepG2-MDR cells[36]. Compared with cyclosporine and verapamil that have been attempted for reversing chemoresistance, bromocriptine has little effect on the cytotoxicity of anticancer drugs.

In our current study, we found that bromocriptine could significantly enhance the uptake of ^99m^Tc-MIBI in hepatocellular carcinoma patients who failed to show any noticeable uptake of ^99m^Tc-MIBI. Our findings suggest that bromocriptine as an inhibitor of *P*-gp activities could be of value as an agent to boost the uptake of ^99m^Tc-MIBI for tumor imaging. It is also tempting to speculate that bromocriptine can be included as part of the therapeutic regimen to overcome chemoresistance of cancer cells. Currently, we are conducting studies on the combination of bromocriptine with other chemotherapeutic agents in postoperative liver cancer patients.
